# Supplementary material for: The Potential of the Fibronectin Inhibitor Arg-Gly-Asp-Ser in the Development of Therapies for Glioblastoma
Source: Int J Mol Sci. 2024 Apr 30;25(9):4910. doi: 10.3390/ijms25094910 (PMC11084566; doi:10.3390/ijms25094910)
Supplement: Supplementary file 1 [file ijms-25-04910-s001.zip › ijms-2951342-supplementary.pdf]

Article - Supplementary Materials

The Potential of the Fibronectin Inhibitor Arg-Gly-Asp-Ser in the Development of Therapies for Glioblastoma

Maria L. Castro-Ribeiro [1,2], Vânia I. B. Castro [1,2], Joana Vieira de Castro [1,2], Ricardo A. Pires [1,2], Rui L. Reis [1,2], Bruno M. Costa [2,3], Helena Ferreira [1,2,*] and Nuno M. Neves [1,2,*]

[1] 3B's Research Group, I3Bs—Research Institute on Biomaterials, Biodegradables and Biomimetics, University of Minho, Headquarters of the European Institute of Excellence on Tissue Engineering and Regenerative Medicine, AvePark, Parque de Ciência e Tecnologia, Zona Industrial da Gandra, 4805-017 Guimarães, Portugal; norcrib@gmail.com (M.L.C.-R.); vaniacastro@i3bs.uminho.pt (V.I.B.C.); joana.castro@i3bs.uminho.pt (J.V.d.C.); rpires@i3bs.uminho.pt (R.A.P.); rgreis@i3bs.uminho.pt (R.L.R.)

[2] ICVS/3B's—PT Government Associate Laboratory, 4710-057/4805-017 Braga/Guimarães, Portugal; bfmcosta@med.uminho.pt

[3] Life and Health Sciences Research Institute (ICVS), School of Medicine, Campus Gualtar, University of Minho, 4710-057 Braga, Portugal

* Correspondence: helenaferreira@i3bs.uminho.pt (H.F.); nuno@i3bs.uminho.pt (N.M.N.)

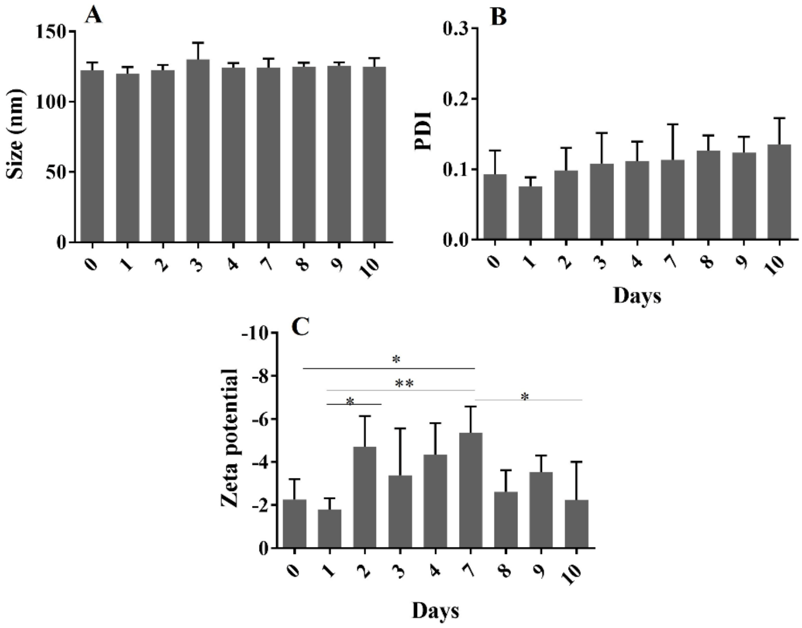

Figure S1. Size (A), PDI (B), and zeta potential (C) of DPPC liposomes for 10 days. The symbol (*) denotes significant differences in the different days: **$p < 0.01$; *$p < 0.05$.

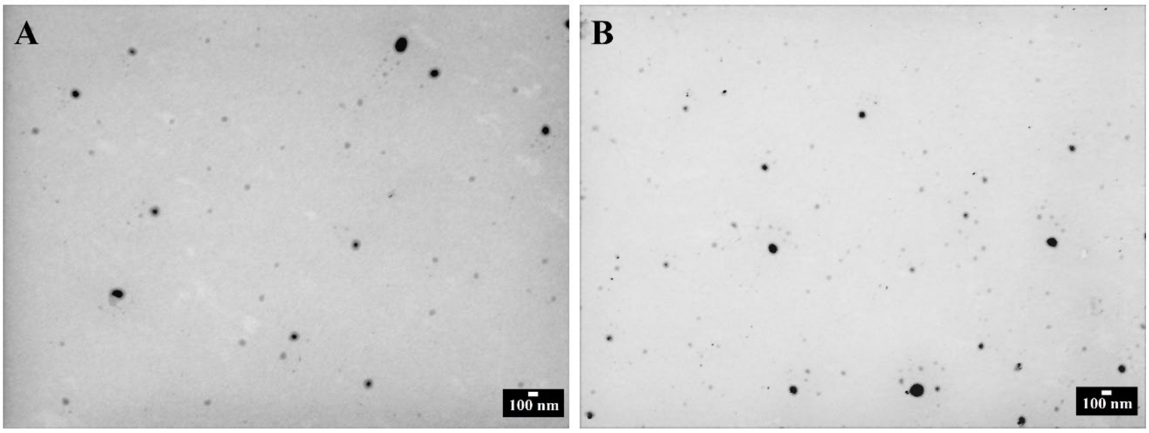

Figure S2. STEM images of empty LUVs (A) or incorporating DOX (B). Scale bar of 100 nm.

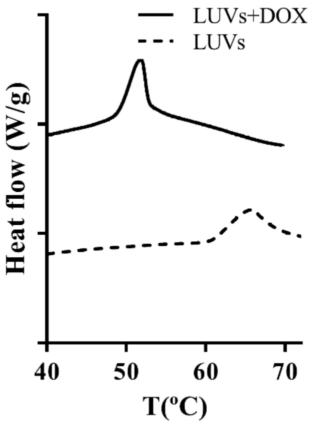

Figure S3. DSC thermogram of freeze-dried empty LUVs and incorporating DOX (LUVs+DOX).

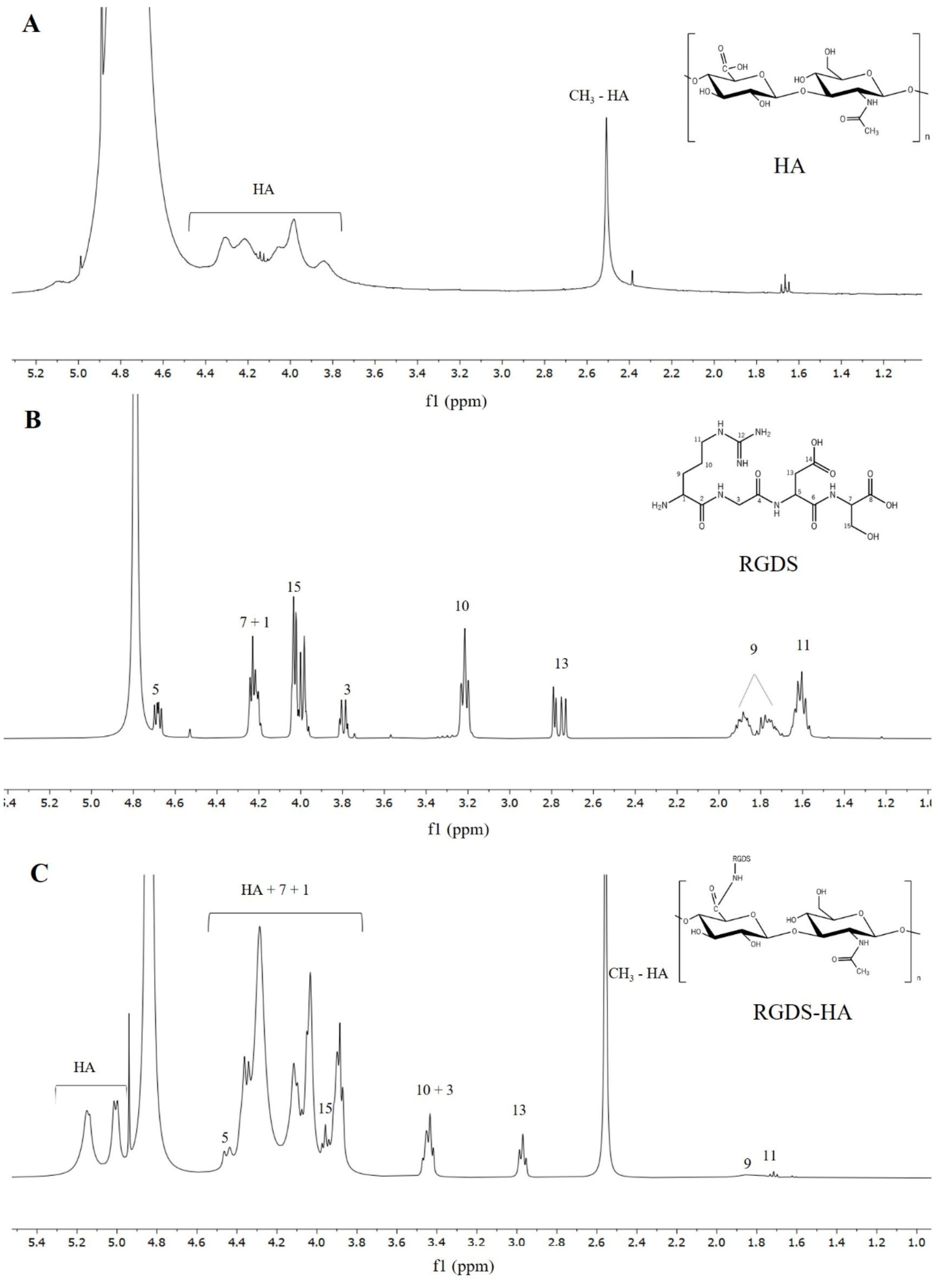

Figure S4. ^{1}H-NMR spectra of HA (A), RGDS (B), and HA-RGDS (C).

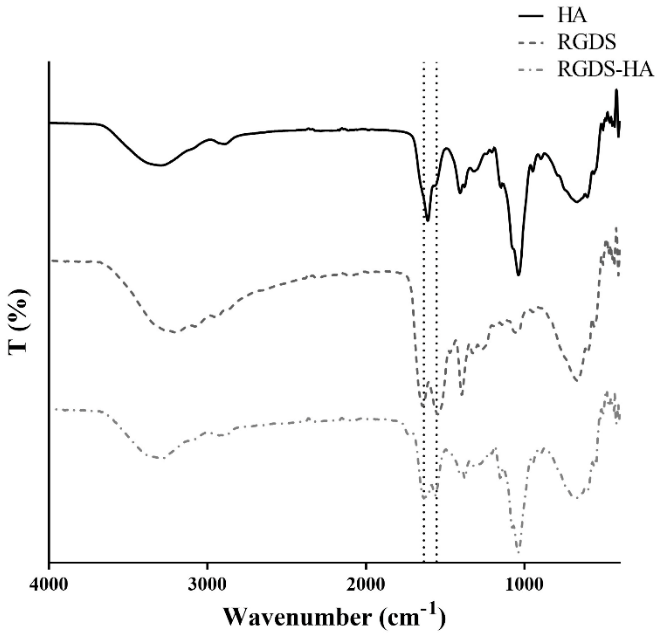

Figure S5. ATR-FTIR spectra of HA, RGDS, and their chemical reaction product (RGDS-HA).

Table S1. Values of elastic (G′) and viscous (G′′) moduli of hydrogels containing 1%, 2.5%, and 5% (w/v) HA at 25 °C, and 37 °C at 1 Hz of frequency.

HA concentration (% w/v)	T (°C)	G′(kPa)	G′′ (kPa)
1	25	0.017±0.007	0.006±0.002
	37	0.021±0.006	0.015±0.006
2.5	25	0.428±0.094	0.292±0.096
	37	0.255±0.092	0.184±0.050
5	25	3.836±0.906	1.506±0,232
	37	2.479±0.670	1.161±0.185

Table S2. Values of elastic (G′) and viscous (G′′) moduli of hydrogels of HA and HA functionalized with RGDS (RGDS-HA) and with 150 μM of LUVs (HA+LUVs and RGDS-HA+LUVs), at 25 °C (A), and 37 °C (B) with 1% strain at 1HZ of frequency.

Formulation	T (°C)	G′(kPa)	G′′(kPa)
HA	25	3.836±0.906	1.506±0,232
	37	2.479±0.670	1.161±0.185
HA+LUVs	25	3.824±1.595	1.527±0.589
	37	2.351±0.851	1.109±0.236
RGDS-HA	25	0.888±0.200	0.530±0.125
	37	0.835±0.091	0.549±0.062
RGDS-HA+LUVs	25	0.784±0.081	0.561±0.060
	37	0.718±0.047	0.540±0.030

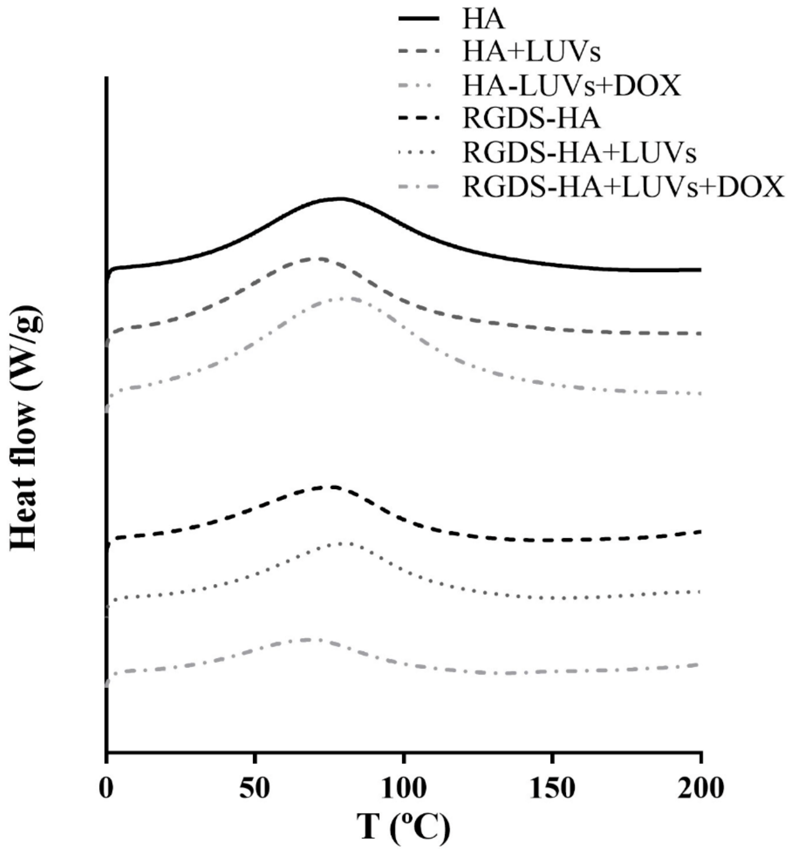

Figure S6. DSC thermograms of HA and after functionalization with RGDS (RGDS-HA) and incorporating LUVs (HA+ LUVs and RGDS-HA+LUVs) and LUVs containing DOX (HA+LUVs+DOX and RGDS-HA+LUVs+DOX).

Table S3. Values of endothermic peaks and ΔH of endothermic events for hydrogels of HA and of HA functionalized with RGDS (RGDS-HA), with LUVs (HA+LUVs and RGDS-HA+LUVs), and with LUVs+DOX (HA+LUVs+DOX and RGDS-HA+LUVs+DOX).

Formulation	Endothermic Peak (°C)	ΔH (J/g)
HA	75.36 ± 2.53	+ 481.53 ± 45.35
HA+LUVs	74.99 ± 4.63	+ 490.16±48.96
HA+LUVS+DOX	78.72 ± 3.11	+ 597.77±74.84
RGDS-HA	77.68 ± 4.26	+ 350.90±40.38
RGDS-HA+LUVs	81.10 ± 1.39	+ 405.37±66.23
RGDS-HA+LUVs+DOX	79.06 ± 8.79	+ 378.10±137.55

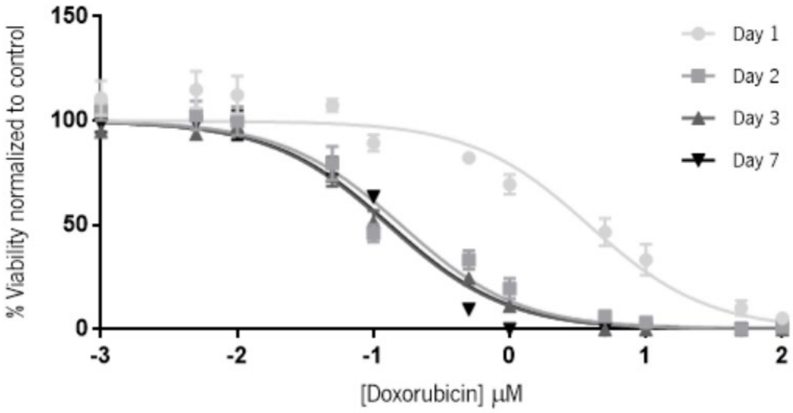

Figure S7. Viability (%) of GBML42 cells incubated with different concentrations of DOX (0.001, 0.005, 0.01, 0.05, 0.1, 0.5, 1, 5, 10, 50, and 100 μM) after 1, 2, 3, and 7 days of culture.

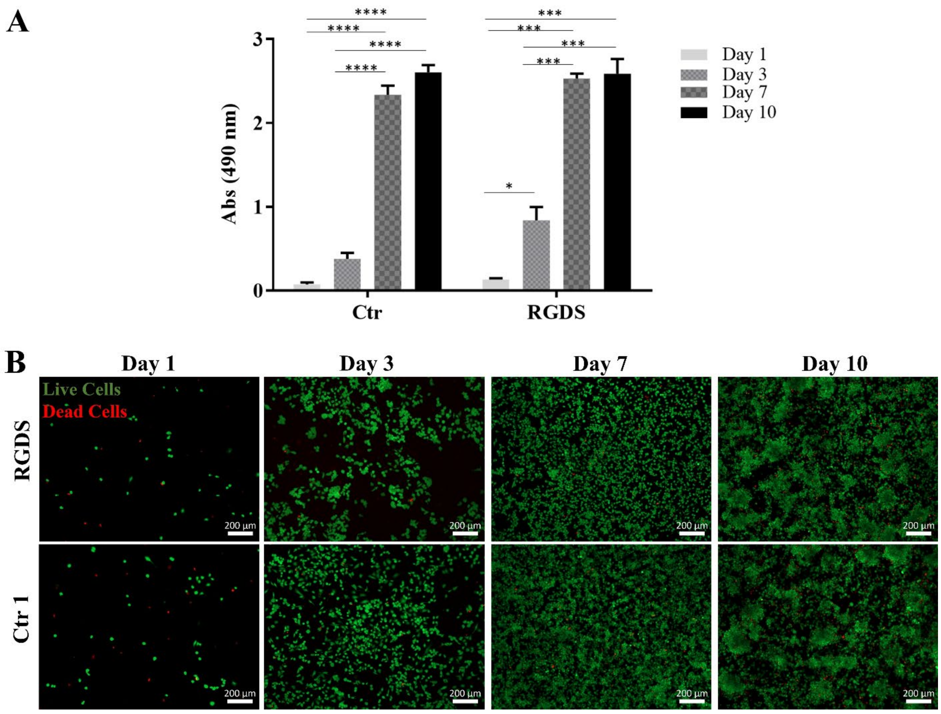

Figure S8. Metabolic activity (A) and fluorescence microscopy images of live/dead staining with Calcein AM/PI (green: live cell; red: dead cell; B) of GBML42 cells in the presence of 500 ng/mL of RGDS after 1, 3, 7, and 10 days of culture. Ctr refers to GBML42 cells cultured only with the medium. The symbol (*) denotes significant differences in the different days for each condition: ****$p < 0.0001$; ***$p < 0.001$; *$p < 0.05$.

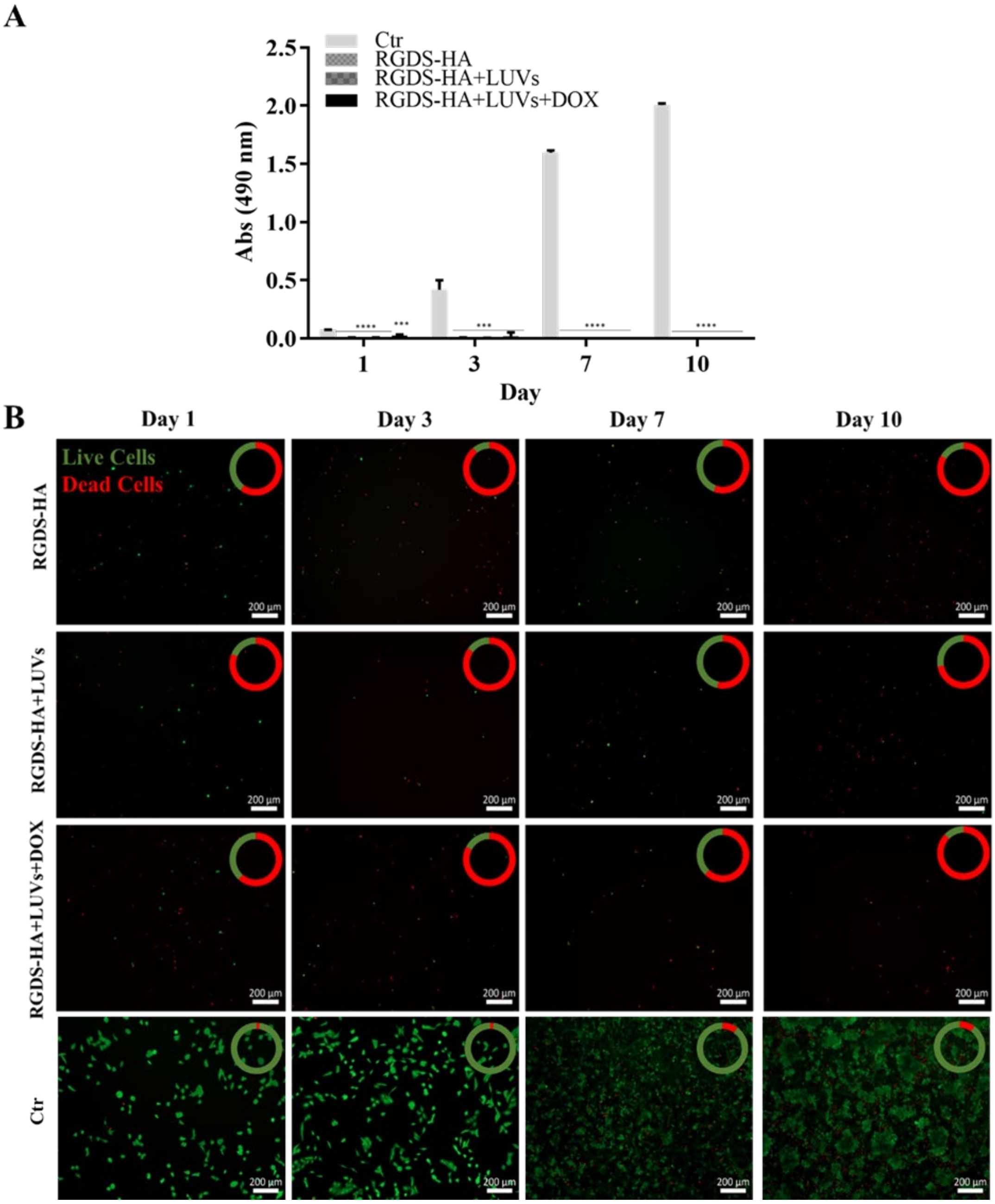

Figure S9. Metabolic activity (A) and fluorescence microscopy images of live/dead staining with Calcein AM/PI (green: live cell; red: dead cell; B) of GBML42 cells cultured on 24-well plates plate (control: Ctr) or seeded on RGDS-functionalized HA (RGDS-HA) without or with 150 µM LUVs (RGDS-HA+LUVs) not or encapsulating 0.1 µM DOX (RGDS-HA+LUVs+DOX). In A, the symbol (*) denotes significant differences versus the Ctr: ****$p < 0.0001$; ***$p < 0.001$.The live/dead images indicate the amount of live cells (green) in comparison to dead cells (red).
